# Supplementary material for: RAC1B Suppresses TGF-β1-Dependent Cell Migration in Pancreatic Carcinoma Cells through Inhibition of the TGF-β Type I Receptor ALK5
Source: Cancers (Basel). 2019 May 17;11(5):691. doi: 10.3390/cancers11050691 (PMC6562819; doi:10.3390/cancers11050691)
Supplement: Supplementary file 1 [file cancers-11-00691-s001.pdf]

cancers

MDPI

*Article*

# RAC1B Suppresses TGF-β1-Dependent Cell Migration in Pancreatic Carcinoma Cells Through Inhibition of the TGF-β Type I Receptor ALK5

**Supplementary Material**

**Hendrik Ungefroren, Hannah Otterbein, Christian Fiedler, Koichiro Mihara, Morley D. Hollenberg, Frank Gieseler, Hendrik Lehnert and David Witte**

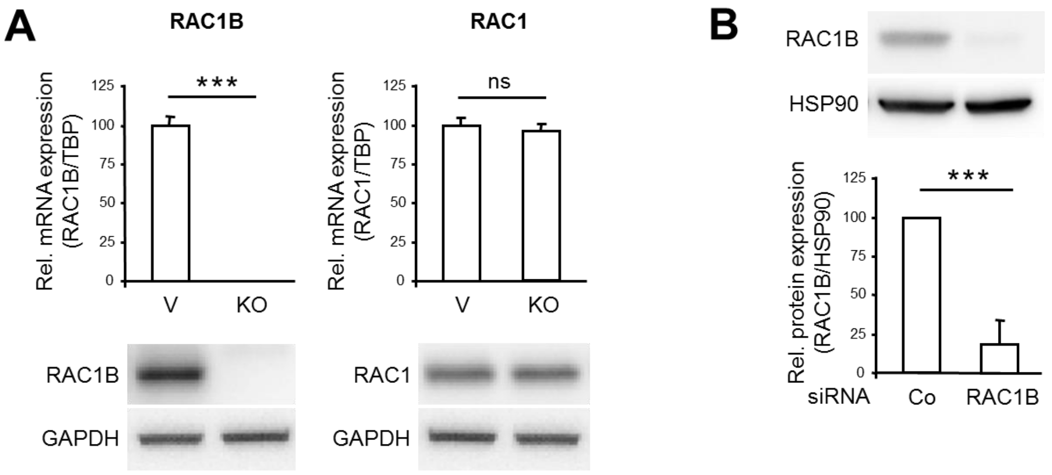


**Figure 1.** Quantification of RAC1B expression in Panc1-RAC1B-KO and Panc1-RAC1B-KD cells. (**A**) Panc1 cells in which exon 3b of *RAC1* had been deleted by CRISPR/Cas technology (Panc1-RAC1B-KO) were subjected to qPCR and immunoblot analysis for RAC1B, and RAC1 as control. The qPCR data (graphs) were normalized to those for TBP and are the means ±SD from three parallel wells. Below the graphs the corresponding immunoblots are shown. GAPDH was used as a loading control. (**B**) Panc1 cells were transiently transfected twice (on two consecutive days) with 50 nM of either control siRNA (Co) or RAC1B siRNA (RAC1B). Forty-eight h after the second round of transfection cells were lysed and subjected to immunoblot analysis for RAC1B, and HSP90 as a loading control. The graph underneath the blot shows quantification from densitometric analyses. Signal intensities for RAC1B were normalized to those for HSP90 and represent the mean ±SD from six independent experiments. The asterisks indicate significance; ns, not significant. For a detailed description of the generation of Panc1-RAC1B-KO and –KD cells see Methods section.

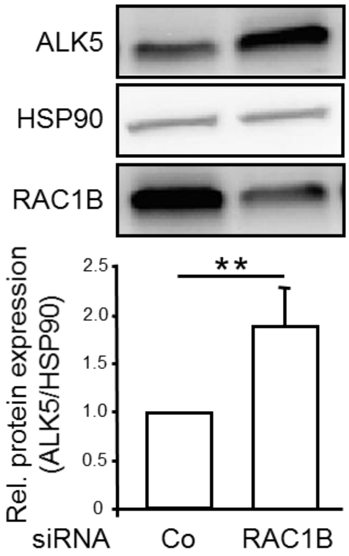


**Figure 2.** Effect of RAC1B KD on ALK5 expression in the PDAC-derived cell line Colo357. Colo357 cells were transfected twice (on two consecutive days) with 50 nM of either irrelevant control siRNA (Co) or siRNA specific for RAC1B (RAC1B). Forty-eight h later cells were subjected to immunoblotting for ALK5, HSP90 as a loading control, and RAC1B as a control for transfection efficiency. The graph underneath the blot shows quantification from densitometric analyses. Signal intensities for ALK5 were normalized to those for HSP90 and represent the mean ±SD from three independent experiments. The asterisks indicate significance (student's *t* test).

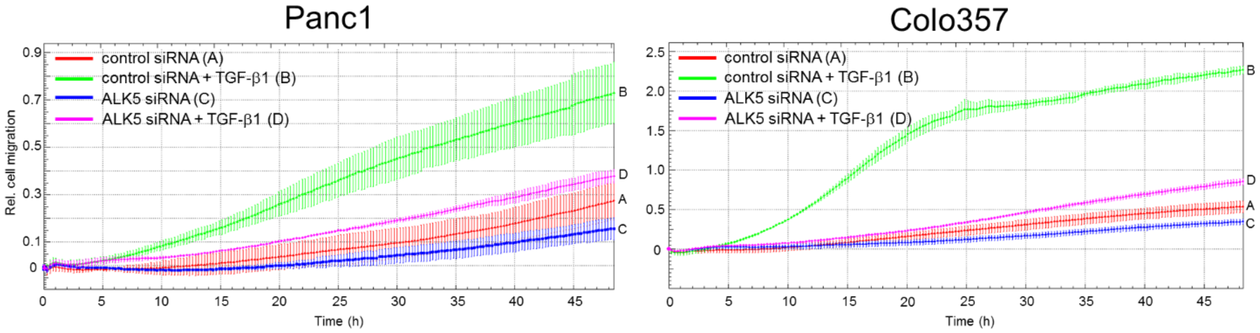


**Figure 3.** Effect of ALK5 KD on TGF-β1-induced migration of Panc1 and Colo357 cells. Panc1 or Colo357 cells were transfected twice with 50 nM of either control siRNA or ALK5 siRNA. Forty-eight h after the second transfection, cells were processed for migration assay on the xCELLigence platform. Immediately before the start of the assay one half of the cells received 5 ng/mL TGF-β1. Data are from one representative experiment and are the mean ±SD from 3–4 wells per condition. Differences between Panc1 cells + ALK5 siRNA + TGF-β1 (magenta curve, tracing D) and Panc1 cells + control siRNA + TGF-β1 (green curve, tracing B) are significant at 07:45 and all later time points. Differences between Colo357 cells + ALK5 siRNA + TGF-β1 (magenta curve, tracing D) and Colo357 cells + control siRNA + TGF-β1 (green curve, tracing B) are significant at 06:30 and all later time points. Successful inhibition of ALK5 protein expression was verified by immunoblotting (not shown). For functional validation of the ALK5 siRNA see Figure 1A.

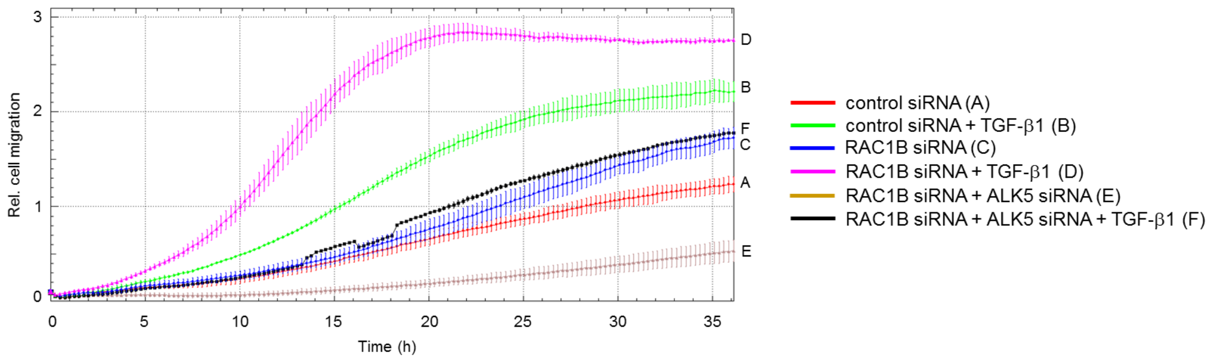


**Figure 4.** Effect of RAC1B KD, ALK5 KD, and combined RAC1B/ALK5 KD on TGF-β1-induced migration of Colo357 cells. Colo357 cells were transfected twice with either 50 nM of control siRNA, 25 nM RAC1B siRNA+ 25 nM control siRNA, or 25 nM RAC1B siRNA + 25 nM ALK5 siRNA. Forty-eight h after the second transfection, cells were processed for migration assay on the xCELLigence platform. Immediately prior to the start of the assay one half of the cells received 5 ng/ml TGF-β1. Data are from one representative experiment and are the mean ± SD from 4 wells per condition. Differences between Colo357 cells + RAC1B siRNA + ALK5 siRNA + TGF-β1 (black curve, tracing F) and Colo357 cells + RAC1B siRNA + TGF-β1 (magenta curve, tracing D) are significant at 04:30 and all later time points. Successful inhibition of RAC1B and ALK5 protein expression was verified by immunoblotting (data not shown).

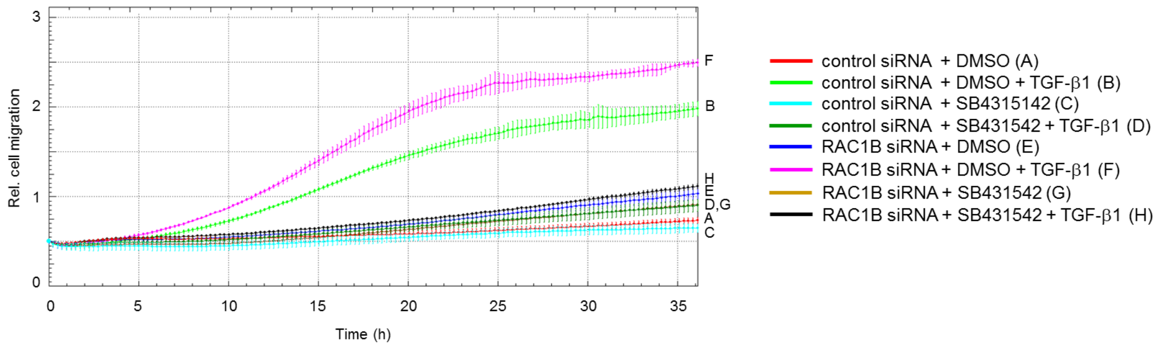


**Figure 5.** Effect of pharmacologic inhibition of the ALK5 kinase activity on TGF-β1 and RAC1B KD-induced migration of Colo357 cells. Colo357 cells were transfected twice, on two consecutive days, with 50 nM of either control siRNA or RAC1B siRNA. Forty-eight h after the second transfection, cells were processed for migration assay on the xCELLigence platform. Immediately prior to the start of the assay one half of the cells received 5 ng/ml TGF-β1 along with either the ALK5 kinase inhibitor SB431542 (5 µM) or solvent (dimethyl sulfoxide, DMSO). Data are from one representative experiment (three performed in total) and are the mean ± SD from 4 wells per condition. Differences between Colo357 cells + RAC1B siRNA + SB431542 + TGF-β1 (black curve, tracing H) and Panc1 + RAC1B siRNA + TGF-β1 (magenta curve, tracing F) are significant at 06:30 and all later time points. Successful inhibition of RAC1B was verified by immunoblotting (data not shown).

**Table S1: Guide sequences used for knockout of *RAC1* exon 3b**

| Designation | Sequence (5'→3') |
|---|---|
| RAC1B CRa | GAGTGTGATAGTTTACCCAC |
| RAC1B CRb | GCAGGCGTTAAGTTCAACGA |
| RAC1B CRc | AGCTCGTCCAAGAATCACCG |
| RAC1B CRd | GTGGGTGCTGCCATGGGAGG |

**Table S2: Primers used for qPCR**

| Designation | Sequence (5'→3') | GenBank accession |
|---|---|---|
| ALK5-sense | GCGACGGCGTTACAGTGTTTCTGC | NM_004612 |
| ALK5-antisense | ATGGTGAATGACAGTGCGGTTGTGG | NM_004612 |
| β-ACTIN-sense | GACGAGGCCCAGAGCAAGAG | NM_001101 |
| β-ACTIN-antisense | ATCTCCTTCTGCATCCTGTC | NM_001101 |
| RAC1B-sense (exon 3b) | GGGGCAAAGACAAGCCGAT | NM_018890 |
| RAC1B-antisense | CTCGGATCGCTTCGTCAAAC | NM_018890 |
| RAC1-sense | AGGCCATCAAGTGTGTGGTG | NM_018890 |
| RAC1-antisense | AGAACACATCTGTTTGCGGAT | NM_018890 |
| TBP-sense | GCTGGCCCATAGTGATCTTT | M55654.1 |
| TBP-antisense | CTTCACACGCCAAGAAACAG | M55654.1 |
| TBP-sense | AACAACAGCCTGCCACCTTA | M55654.1 (Figure S1) |
| TBP-antisense | GCCATAAGGCATCATTGGAC | M55654.1 (Figure S1) |
